# Discovery of prostate specific antigen pattern to predict castration resistant prostate cancer of androgen deprivation therapy

**DOI:** 10.1186/s12911-016-0297-0

**Published:** 2016-07-18

**Authors:** Yejin Kim, Yong Hyun Park, Ji Youl Lee, In Young Choi, Hwanjo Yu

**Affiliations:** 1 Department of Creative IT Engineering, POSTECH, Pohang, South Korea; 2 Department of Urology, Seoul St. Mary’s Hospital, Seoul, South Korea; 3 Department of Medical Informatics, The Catholic University of Korea College of Medicine, Seoul, South Korea

**Keywords:** Prostate specific antigen, Longitudinal biomarker, Frequent sequential pattern mining, Prediction

## Abstract

**Background:**

Prostate specific antigen (PSA) is an important biomarker to monitor the response to the treatment, but has not been fully utilized as a whole sequence. We used a longitudinal biomarker PSA to discover a new prognostic pattern that predicts castration-resistant prostate cancer (CRPC) after androgen deprivation therapy.

**Methods:**

We transformed the longitudinal PSA into a discrete sequence, used frequent sequential pattern mining to find candidate patterns from the sequences, and selected the most predictive and informative pattern among the candidates.

**Results:**

Patients were less likely to be CRPC if, after PSA values reach nadir, the PSA decreases more than 0.048 ng/ml during a month, and the decrease occurs again. This pattern significantly increased the accuracy of predicting CRPC by supplementing information provided by existing PSA patterns such as pretreatment PSA.

**Conclusions:**

This result can help clinicians to stratify men by the risk of CRPC and to determine the patient that needs intensive follow-up.

## Background

Prostate cancer has been the most common cancer in men worldwide [1–3]; it accounted for 27 % of new cancer cases in 2014 [3]. Androgen deprivation therapy (ADT) is the primary treatment of metastatic prostate cancer. ADT is conducted by suppressing androgens by castration, inhibiting the action of androgen using competing compounds known as anti-androgens, or by combining these treatments. Unfortunately, some patients proceed to castration-resistant prostate cancer (CRPC), whereas others retain hormone-sensitive prostate cancer (HSPC). For those who will be possibly endangered to CRPC, intensive follow-up and additional systematic therapies are required. Thus clinicians must assess the risk of progression to CRPC.

Prostate specific antigen (PSA) has been an important biomarker for diagnosis and prognosis of ADT. PSA level is measured during follow-up to monitor the response to the treatment. Generally, PSA level decreases after ADT begins, reaches the lowest level (nadir), then stabilizes for some period. If the cancer develops, PSA level increases. PSA has been used in therapeutic decision making by stratifying the risk of development to CRPC [4]. Characteristics of PSA variation are summarized as patterns such as pretreatment PSA level, nadir, time to nadir, and doubling time; these patterns have clinical significances as prognostic factors to predict CRPC [5–10]. However, the accuracy of these patterns as predictors is still unclear. They are computed based on only one or two PSA values before or around nadir even though PSA accumulates consistently after the treatment.

Patterns generated from a fully-utilized PSA sequence may increase the accuracy of predicting CRPC. Although the latter parts of PSA accumulation reflect the progression to CRPC [4], they have been discarded for three reasons: (1) Collection of PSA data from electronic medical records has been limited [11]; (2) Computing the characteristic patterns with the whole PSA sequence is more complicated than with one or two representative values; and (3) The relationship between PSA after nadir and CRPC has not been fully quantified. However, the patterns of PSA after nadir can provide insight into this relationship.

Thus we aim to exploit longitudinal PSA data to discover a new prognostic pattern that predicts CRPC after ADT, and to demonstrate clinical significance of the new pattern. We will compare this pattern with existing patterns.

## Methods

We described a framework that discovers the prognostic pattern from the longitudinal PSA. This framework consists of three parts: transformation, pattern mining, and pattern selection.

### Materials

**Patients.** We exploited data in electronic medical records (EMRs) that include longitudinal PSA level and other clinical variables. The EMR data were from an observational longitudinal database at Seoul, St. Mary Hospital, Korea; the database has been described in detail previously [12]. Among 1068 men diagnosed from January 2006 to June 2012 at our institution, 458 were treated as ADT. We only included 370 men who had not received any other treatment such as radical prostatectomy or radiation therapy and for whom PSA level data were available.

**Longitudinal PSA.** Each patients had a set of longitudinal PSA values that were recorded during follow-up every one to six months. The time to nadir has been investigated as the primary time point at which the kinetics of PSA level changes [6]; thus we separated the longitudinal PSA sequences into before and after nadir. Among the total of 2883 PSA values, 1238 were before nadir and 1645 were after nadir. We excluded PSA after CRPC because we should predict CRPC before it occurs. Some patients had only before or after nadir. After separation, 261 men had PSA after nadir, and 306 men had PSA before nadir.

**Other variables.** Patients had 14 demographic and clinical features: Age, laboratory results of Alb, Plt, Hb, Ca; medication information on intermittent treatment, drug order; bone metastasis, clinical stage; Gleason score, MRI prostate volume, pretreatment PSA, nadir, time to nadir. Patients also had two outcome variables: dichotomous factor for CRPC occurrence, and the time to CRPC. The CRPC variables were determined after being reviewed by the single urologist (Y.H.P.). Missing values were imputed using random survival forest [13]. To provide clinical background profiles of patients, the representative characteristics of the patients that have PSA after nadir were evaluated. Existence of bone metastasis can be cause of CRPC [14], and CRPC patients are more likely to have high Gleason score [15]. The *p*-value of the most representative features to predict CRPC by univariate Cox regression was assessed (Table 1).

**Table 1:** Characteristics and *p*-value of patients that have PSA after nadir

	Total	CRPC	*p*-value
Number of patients	261	96	-
Mean follow-up ± s.d.	38.7 ± 3.5	15.8 ± 2.4	-
Mean age ± s.d.	74 ± 0.9	75.2 ± 1.7	0.011
Mean time to nadir ± s.d.	10.3 ± 1.3	8.9 ± 1.9	0.001
Mean pretreatment PSA ± s.d.	119.4 ± 39.4	151.5 ± 80.3	0.054
Mean PSA nadir ± s.d.	12.8 ± 11.7	6.6 ± 4.2	0.142
Bone metastasis			
Yes	41	15	0.490
No	220	79	
Gleason score			0.117
≤ 6	59 (22.6 %)	20 (21.2 %)	
7	72 (27.5 %)	21 (22.3 %)	
≥ 8	130 (49.8 %)	53 (56.3 %)	
ADT type			
Leuprin only	154	44	0.012
Zoladex only	36	10	
Leuprin → Zoladex	9	6	
Zoladex → Leuprin	49	27	
Anti-androgen only	13	7	

### Transformation

#### PSA velocity

We first converted PSA level to PSA velocity (PSAV) [ng/(ml · mo)]:

1 $$ PSAV = \frac{{PSA}_{t_{2}}-{PSA}_{t_{1}}}{t_{2} - t_{1}},  $$

where ${PSA}_{t_{i}}$ is PSA [ng/ml] at time *t*_*i*_ [mo] [16]; so PSA sequence was converted into PSAV sequence, which can capture directions and the amount of PSA change. PSAV ≥0 and ≤0 referred to increasing and decreasing PSA level per month, respectively. PSAV is sometimes expressed as logarithm [17], but we did not log-transform PSA because log(*PSA*) change means relative change (i.e. multiple of previous PSA) rather than absolute change. We discriminated small change from large change when the ratio of two PSA values was the same. For example, PSA changes from 0.003 to 0.001 and from 30 to 10 have the same log(*PSA*) changes, but different PSAV.

#### Discretization

PSAV was abstracted using discretization methods because continuous PSAV contains noise that might reduce its generalizability. We denoted PSAV state as the discretized PSAV; so PSAV sequence was converted to PSAV state sequence. Two discretization methods were used: equal-frequency binning and entropy-based discretization. We used both methods and compared them to avoid biased discretization split-points.

- Equal-frequency binning is an unsupervised discretization technique that splits continuous variables into a specified number of bins to have equal frequency. We set bin size to five. These PSAV states were labeled as Low (*L*_*q*_), Medium low (*ML*_*q*_), Medium (*M*_*q*_), Medium high (*MH*_*q*_), and High (*H*_*q*_). This method did not distinguish PSAV of CRPC from PSAV of HSPC.
- Entropy-based discretization is a supervised discretization technique that finds split-points with minimum entropy, and recursively partitions the intervals until a stopping criterion is met [18]. PSAV values were separated into three PSAV states, which were labeled as Low (*L*_*e*_), Medium (*M*_*e*_), and High (*H*_*e*_). Because entropy is increased when PSAV of CRPC and PSAV of HSPC are mixed, this method generated PSAV states so that PSAV values from CRPC and HSPC belonged to distinct PSAV state as much as possible.

**Fig. 1:**
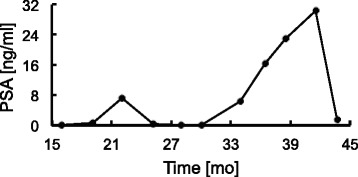
PSA sequence. Example of transformation based on after-nadir longitudinal PSA by entropy-bases discretization

**Fig. 2:**
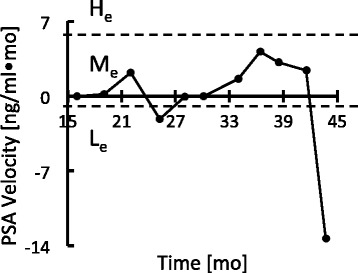
PSAV sequence and PSAV state. Example of transformation based on after-nadir longitudinal PSA by entropy-bases discretization. PSA sequence (Fig. 1) was transformed to PSAV sequence (Fig. 2), then PSAV state (*L*_*e*_,*M*_*e*_,*H*_*e*_) sequence. Dashed lines: borders between PSAV states (labeled)

As an example of the transformation using after-nadir longitudinal PSA by entropy-based discretization we consider the PSA sequence (Figs. 1 and 2) from a patient treated with two medications (Zoladex and Leuprin) intermittently. PSA level reached nadir at 15 months after treatment began (Fig. 1). The PSA values were converted into PSAVs (Fig. 2), and then PSAVs were assigned to one of the PSAV states (i.e. *L*_*e*_, *M*_*e*_, *H*_*e*_) that separate PSAVs by the dashed lines (Fig. 2). The interval of PSAV states is computed by entropy-based discretization. PSAV values at (*∞*,−1),[−1,6), and [6,*∞*) are discretized as *L*_*e*_,*M*_*e*_, and *H*_*e*_, respectively. Consequently, the PSA sequence becomes the PSAV state sequence *M*_*e*_→*M*_*e*_→*M*_*e*_→*L*_*e*_→*M*_*e*_→*M*_*e*_→*M*_*e*_→*M*_*e*_→*M*_*e*_→*M*_*e*_→*L*_*e*_.

### Pattern mining

To fully utilize longitudinal PSA data, we split PSAV state sequence into before- and after-nadir PSAV state sequences, and investigated the whole PSAV state sequence at the same time. We then used the frequent sequential pattern mining (FSPM) method to find new prognostic patterns. This method is the most widely used method for a set of discrete sequences. This method is also more computationally advantageous for a set of short and single sequences than are other methods that were mostly devised to analyze heterogeneous and large-scale data [19–22]. The PSAV state sequences were also short due to the short follow-up periods. We used FSPM to find candidate prognostic PSA patterns.

Particularly we used PrefixSpan algorithm for FSPM, which is a pattern-growth approach that builds prefix patterns that concatenate with suffix patterns to find frequent patterns [18, 23]. For examples, let assume that we have PSAV state sequences in Table 2 and minimal frequency of 0.1. We began with length-one prefix. The number of instance of length-one sequential patterns is L: 5, ML: 4, M: 6, MH: 1, H: 2. We discarded MH with frequency (=1/18) < 0.1. We then divided the search space with each prefix and searched sequential patterns starting with the prefix. We listed up the subset of PSAV state sequences starting with the prefix and discarded PSAV state sequence with frequency < 0.1. We repeated these process until the prefix becomes the whole PSA state sequence.

**Table 2:** Example of PrefixSpan

	PSAV state sequences
1	*L*→*L*→*L*→*M* *H*→*M*
2	*L*→*M*→*M* *L*→*L*
3	*H*→*M* *L*→*M*→*M*→*M* *L*
4	*H*→*M*→*L*→*M*→*M* *L*→*M*

We restricted the patterns to have a support ≥0.3 to ensure the enough frequency, and a length ≤3 to avoid over-fitting. The discovered set of candidate patterns were the subset of PSAV state sequences. The patterns were time-ordered but not always consecutive.

### Selection

#### Predictive pattern selection

To select the predictive patterns among the candidates set that were generated from FSPM, we evaluated the accuracy by measuring the area under the receiver-operating characteristic curve (AUC) and Harrell’s concordance index (C-index) [24]. Each candidate pattern *p* was used as the predictor of CRPC because, by contraposition, if HSPC patients have a pattern *p*, a patient without the pattern *p* would be CRPC, and vice versa. We used baseline set containing the 14 demographic and clinical features that were extracted from the EMRs. We added each candidate pattern *p* to the baseline *B* (i.e. *B*∪*p*) to evaluate the pattern *p*. We evaluated the discriminative power of the pattern *p* using

2 $$ \bigtriangleup \text{AUC}=\text{AUC}(B \cup p) - \text{AUC}(B),  $$

and

3 $$ \bigtriangleup \text{C-index} = \text{C-index}(B\cup p) - \text{C-index}(B)  $$

where AUC(*X*) denotes AUC of a logistic regression to predict CRPC using dataset *X*, and C-index(*X*) denotes the C-index of a Cox regression to predict time to CRPC using dataset *X*. They represent the net increase in AUC and C-index compared to the baseline. Ten-fold cross validation was used. A paired *t*-test with 95 % confidence level was conducted to identify patterns that increase the AUC and C-index significantly; patterns that had *p*-value ≤0.05 were excluded. The remaining significantly predictive patterns among the candidate set became the final candidate set from which the last pattern was selected.

#### Informative pattern selection

Among the significantly predictive patterns, we chose the most informative pattern. We preferred specific and rare patterns to broad and prevalent ones if the patterns have similar accuracy, because the former pattern provides relatively more information than the latter one. We formulated the relative information as follows:

##### **Lemma1**.

Let *p*_1_,*p*_2_ denote patterns that the prediction accuracy are not significantly different, and let *I*(*p*) denote the relative amount of information expressed by pattern *p*. Then *I*(*p*_2_)≤*I*(*p*_1_) if

1. *p*_2_ is a sub-pattern of *p*_1_ or
2. The interval of *p*_1_ is a subset of interval of *p*_2_ or
3. The frequency of *p*_1_ is smaller than frequency of *p*_2_.

Cases 1 and 2 indicate that all patients that have *p*_1_ also have *p*_2_; thus *p*_1_ is more specific than *p*_2_. For example, *p*_2_ is the sub-pattern of *p*_1_ if *p*_1_=*L*→*L*, *p*_2_=*L* because L is a sub-pattern of *L*→*L* (Case 1). When *p*_1_=*L*_*e*_, *p*_2_=*L*_*q*_ where *L*_*e*_ has the interval of PSAV ≤−0.048, and *L*_*q*_ has the interval PSAV ≤−0.005, then *p*_1_ is more specific than *p*_2_ (Case 2). Case 3 implies that *p*_1_ occurs more rarely than *p*_2_. If *p*_1_ is rare than *p*_2_ although *p*_1_ and *p*_2_ have similar prediction accuracy, it means that the amount of information that *p*_1_ carries per instance. For example, if *p*_1_=*L*_*e*_, *p*_2_=*M*_*q*_ where the frequency of *p*_1_ is 14.4 %, and the frequency of *p*_2_ is 59.3 %, then *p*_1_ is more informative than *p*_2_ because *p*_1_ is more rare than *p*_2_ in spite of the same prediction accuracy. We compared the amount of information using Lemma 1, and selected from the candidate set the final prognostic pattern that has the largest amount of information.

#### Comparison

We compared the progression to CRPC of the final pattern with that of pretreatment PSA, nadir, and time to nadir, which are known as the prognosis factors of CRPC. We computed the log-rank statistics of Kaplan-Meier analysis to test survival difference between patients with and without the pattern. The thresholds of pretreatment, nadir, and time to nadir were 100 ng/ml, 0.2 ng/ml, and 12 months, respectively [6].

#### Software

We used R3.0.3 [25] with two packages: survival [26, 27] for the significance test, Cox regression and log-rank test; and randomSurvivalForest [13, 27] for the imputation. We also used JAVA API, SPMF [28] to implement FSPM.

## Results

We separated the longitudinal PSA data into before and after nadir. For the after-nadir dataset, we had 261 patients (HSPC: 167, CRPC: 94), and the mean follow-up time was 38.7 ± 3.5 months; the mean time to CRPC was 15.8 ± 2.4 months. Median PSAV was 0.022 ng/(ml · mo) (from -1521 to 2091). (Analysis of the before-nadir data did not reveal any prognostic patterns (Appendix Appendix A: Results of before-nadir PSA values)).

We discretized the continuous PSAV values into five and three PSAV states by equal-frequency binning (Table 3) or entropy-based discretization (Table 4). Equal-frequency binning generated PSAV states of which frequency was evenly distributed over the five states. Entropy-based method generated three PSAV states; 85 % were *M*_*e*_, 14 % were *L*_*e*_, and 1 % were *H*_*e*_. *M*_*e*_ occurred in both CRPC and HSPC, *L*_*e*_ occurred only in HSPC, and *H*_*e*_ occurred only in CRPC.

**Table 3:** Discretization to PSAV state after nadir by equal-frequency binning

PSAV state	Interval	
	Frequency [%]	Value [ng/(ml ·mo)]
*L*_*q*_	(0, 20]	(, −0.005]
*M* *L*_*q*_	(20, 40]	(−0.005, 0.005]
*M*_*q*_	(40, 60]	(0.005, 0.068]
*M* *H*_*q*_	(60, 80]	(0.068, 0.454]
*H*_*q*_	(80, 100]	(0.454,)

*L*_*q*_= Low, *M* *L*_*q*_= Medium low, *M*_*q*_= Medium, *M* *H*_*q*_= Medium high, *H*_*q*_= High

**Table 4:** Discretization to PSAV state after nadir by entropy-based discretization

PSAV state	Interval	
	Frequency [%]	Value [ng/(ml ·mo)]
*L*_*e*_	(0, 14.1]	(, −0.048]
*M*_*e*_	(14.1, 99.2]	(−0.048, 5.43]
*H*_*e*_	(99.2, 100]	(5.43,)

*L*_*e*_= Low, *M*_*e*_= Medium, *H*_*e*_= High

We found the candidate patterns from the set of after-nadir PSAV state sequences. Among the after-nadir 261 patients’ sequences, we found 13 HSPC and 3 CRPC frequent patterns by equal-frequency binning (Table 5); and 6 HSPC and 2 CRPC frequent patterns by entropy-based discretization (Table 6). The PSAV state sequences from CRPC were rather uncommon among them; thus we could not find many frequent patterns from CRPC. In contrast, the PSAV state sequences from HSPC contained patterns that occurred repeatedly.

**Table 5:** After-nadir candidate patterns by equal-frequency binning

Pattern (support)					
HSPC			CRPC		
*L*_*q*_	(0.51),	*L*_*q*_→*L*_*q*_	(0.34)	*M*_*q*_	(0.45)
*M* *L*_*q*_	(0.54),	*M*_*q*_→*L*_*q*_	(0.31)	*M* *H*_*q*_	(0.43)
*M*_*q*_	(0.54),	*M*_*q*_→*M*_*q*_	(0.35)	*H*_*q*_	(0.36)
*M* *H*_*q*_	(0.54),	*M* *H*_*q*_→*L*_*q*_	(0.40)		
*H*_*q*_	(0.48),	*M* *H*_*q*_→*H*_*q*_	(0.31)		
		*M* *H*_*q*_→*M* *H*_*q*_	(0.36)		
		*H*_*q*_→*L*_*q*_	(0.34)		
		*H*_*q*_→*H*_*q*_	(0.32)		

*L*_*q*_,*M* *L*_*q*_,*M*_*q*_,*M* *H*_*q*_,*H*_*q*_= PSAV state (Table 3)

**Table 6:** After-nadir candidate patterns by entropy-based discretization

Pattern (support)			
HSPC		CRPC	
*L*_*e*_	(0.49)	*M*_*e*_	(0.88)
*M*_*e*_	(0.95)	*M*_*e*_→*M*_*e*_	(0.55)
*L*_*e*_→*L*_*e*_	(0.33)		
*L*_*e*_→*M*_*e*_	(0.38)		
*M*_*e*_→*L*_*e*_	(0.49)		
*M*_*e*_→*M*_*e*_	(0.81)		

*L*_*e*_,*M*_*e*_= PSAV state (Table 4)

We computed the △AUC and △C-index when each candidate pattern was added to the baseline, and checked the significances of the △AUC and △C-index by calculating the *p*-values of paired *t*-tests. Five patterns from HSPC and the after-nadir were predictive in that they increased the AUC and C-index significantly (Table 7), but none of the patterns from CRPC or the before-nadir were predictive. The two equal-frequency binning patterns were observed: (1) *L*_*q*_: PSA decline ≥0.005 ng/ml per month after nadir, (2) *L*_*q*_→*L*_*q*_: two PSA declines ≥0.005 ng/ml per month after nadir; they showed the AUC of 0.81 and C-index of 0.78 – 0.79. The two entropy-based discretization patterns with *L*_*e*_ were observed: (1) *L*_*e*_: PSA decline ≥0.048 ng/ml per month after nadir, (2) *L*_*e*_→*L*_*e*_: two PSA declines ≥ 0.048 ng/ml per month after nadir; they showed the AUC of 0.81 – 0.82 and C-index of 0.77 – 0.81. One entropy-based discretization pattern with *M*_*e*_ before *L*_*e*_ was observed; this was *M*_*e*_→*L*_*e*_: PSA decline from 0.048 to 5.43 ng/ml per month followed by PSA decline ≥ 0.048 ng/ml per month after nadir; this pattern showed the AUC of 0.84 and C-index of 0.81.

**Table 7:** Mean and s.d. of logistic regression and Cox regression when each candidate pattern is added to baseline

Pattern	Logistic regression		Cox regression	
	Mean AUC	s.d.	Mean C-index	s.d.
Baseline	0.6951	0.0686	0.6898	0.0429
*L*_*q*_	0.8102	0.0549	0.7938	0.0323
*L*_*q*_→*L*_*q*_	0.8110	0.0500	0.7815	0.0384
*L*_*e*_	0.8103	0.0665	0.8090	0.0442
*L*_*e*_→*L*_*e*_	0.8245	0.0352	0.7733	0.0623
*M*_*e*_→*L*_*e*_	0.8446	0.0459	0.8174	0.0411

The most informative pattern among the five predictive patterns was *L*_*e*_→*L*_*e*_. Because the AUC and C-index among the predictive patterns were not significantly different, we compared the relative amount of information using Lemma 1 regardless of AUC and C-index. By Lemma 1.1,

4 $$ \begin{aligned} I(L_{e}) &\leq I(L_{e} \rightarrow L_{e}), \\ I(L_{e})&\leq I(M_{e} \rightarrow L_{e}), \\ I(L_{q})&\leq I(L_{q} \rightarrow L_{q}). \end{aligned}  $$

We then had *I*(*L*_*e*_→*L*_*e*_),*I*(*M*_*e*_→*L*_*e*_),and *I*(*L*_*q*_→*L*_*q*_) after excluding patterns with small amounts of information. By Lemma 1.2,

5 $$ I(L_{q} \rightarrow L_{q})\leq I(L_{e} \rightarrow L_{e}).  $$

Finally, we compared *I*(*L*_*e*_→*L*_*e*_) and *I*(*M*_*e*_→*L*_*e*_) by Lemma 1.3,

6 $$ I(M_{e} \rightarrow L_{e})\leq I(L_{e} \rightarrow L_{e}).  $$

Thus the final pattern was *L*_*e*_→*L*_*e*_.

**Fig. 3:**
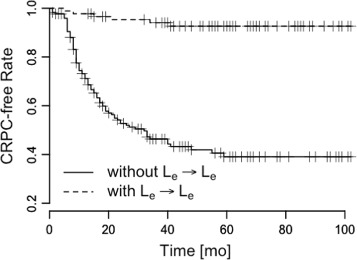
Pattern *L*_*e*_→*L*_*e*_. Time vs. CRPC-free rate to measure survival difference. *p*≤0.001

**Fig. 4:**
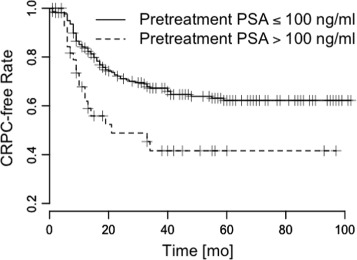
Pretreatment PSA. Time vs. CRPC-free rate to measure survival difference. *p*=0.003

**Fig. 5:**
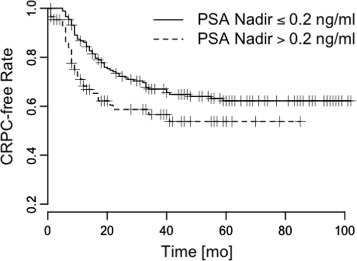
PSA nadir. Time vs. CRPC-free rate to measure survival difference. *p*=0.031

**Fig. 6:**
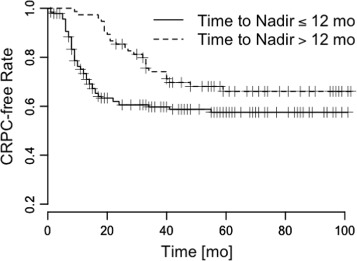
Time to PSA nadir. Time vs. CRPC-free rate to measure survival difference. *p*=0.021

We conducted a Kaplan-Meier analysis of the pattern *L*_*e*_→*L*_*e*_ and the other PSA patterns, and found that patients with *L*_*e*_→*L*_*e*_ showed slow progressions to CRPC, and that patients without *L*_*e*_→*L*_*e*_ showed fast progressions to CRPC (Figs. 3, 4, 5 and 6). The log-rank statistics of all PSA patterns had *p*-values ≤ 0.05. When compared with other PSA patterns, this pattern *L*_*e*_→*L*_*e*_ had comparable prognostic power.

## Discussion

The objective of this study was to exploit the longitudinal measurements of PSA to discover a new prognostic pattern that predicts CRPC. The results of this study demonstrated that ADT patient is more likely to retain HSPC if, after PSA values reach nadir, PSA level decreases more than 0.048 ng/ml during a month, then the decrease occurs again; thus two PSA declines ≥ 0.048 ng/ml per month after nadir could be the prognostic pattern. This pattern was significantly related to the survival time to CRPC.

This finding has not been described in previous research on how different forms of PSA kinetics are associated with prognosis [5–10]. The most representative PSA prognostic patterns are pretreatment PSA, nadir, time to nadir or doubling time. Previous studies did not investigate all available PSA values, but instead used only one or two PSA values before or around nadir; thus the predictive value of PSA change during ADT was not clearly evaluated. In contrast, our pattern was computed from the whole PSA sequence, including even the latter parts. Although the initial response to ADT has important clinical significance, the subsequent response that is inferred from PSA after nadir might also contain information that can be used to predict CRPC. Incorporating PSA level as the sequence might enable us to understand the response to ADT more specifically.

We found that the two substantial declines after PSA nadir ensured sensitivity to ADT. The concept of the two substantial declines after PSA nadir can be confusing for clinicians, because nadir PSA means the lowest PSA value around a given point of observation. The occurrence of two substantial declines after PSA nadir implies that PSA fluctuates after nadir. PSA might increase due to some reasons such as intermittent treatment, and decline as sensitively reacting to ADT. We can further infer that the main difference between HSPC and CRPC is on whether PSA level fluctuates as the response to ADT.

The importance of this study is that the PSA decline after nadir helps to stratify men by the risk of CRPC and to determine the patient population that needs intensive follow-up. Risk assessment of the disease progression to CRPC has been based on the early PSA values, (i.e. before or around nadir) and has been limited to measuring the initial response. PSA after nadir has been neglected due to the complicated nature of computation, but we demonstrated that considering the after-nadir PSA pattern significantly increased the accuracy of the risk assessment by supplementing the early risk assessment obtained using the before-nadir PSA. Thus we can easily identify high-risk men who need in-depth follow-up. Therapeutic decision-making based on appropriate risk stratification enables clinicians to use clinical resource effectively.

This study has two main limitations. The first limitation is that the PSA decline pattern may occur at any time after nadir, so clinicians must wait until the pattern occurs, which must occur after the nadir. This means that clinicians must wait a long time to check whether PSA level declines; this delay is a disadvantage because rapid risk assessment is preferable when designing therapies for high-risk patients. However, FSPM with time constraints can solve this problem [18]. The time-gap between the PSAV states in the discovered pattern can be restricted. The PSAV states that occur within a specified gap reduce the time required to detect the occurrence than PSAV states without the gap. The second limitation is that analysis of the PSA decline pattern was focused on predicting HSPC. The median time to CRPC was only 15.8 months, whereas the median follow-up of all population was 38.7 months. The PSAV sequence from CRPC was not long enough to discover meaningful patterns, so most frequent patters were from HSPC. We predicted CRPC indirectly by predicting HSPC using the PSA decline pattern. A prognostic pattern that occurs frequently in CRPC can help detect CRPC directly, and this prognostic pattern from CRPC can be discovered if the quantity of data is increased and the follow-up time is extended.

## Conclusions

This study discovered a prognostic PSA pattern that predicts CRPC for ADT using FSPM, and demonstrated the clinical significance of the pattern. A patient in which PSA declined twice by ≥ 0.048 ng/ml per month after nadir was predicted to retain HSPC, and a patient in which these declines did not occur was predicted to develop CRPC; the prediction had the AUC of 0.82 if the pattern was combined with pretreatment PSA, nadir, and time to nadir. These results can help risk stratification of ADT patients.

## Appendix A: Results of before-nadir PSA values

For the before-nadir dataset, we had 306 patients (HSPC: 233, CRPC: 73), and the mean follow-up time was 37.7 ± 3.5 months; the mean time to CRPC was 17.5 ± 0.3 months. Median PSAV was -0.12 ng/(ml · mo) (from -1917 to 1686). After discretization, equal-frequency binning gave five discrete PSAV states (Table 8); but the entropy-based discretization could not be applied because the PSAV distributions of CRPC and HSPC were too similar. Among the 306 patients’ PSAV state sequences, we discovered 6 HSPC and 5 CRPC frequent patterns from equal-frequency binning (Table 9). We computed the AUC and C-index when each frequent pattern is added to the baseline, but we could not find patterns that increase AUC and C-index significantly.

**Table 8:** Discretization to PSAV state before nadir by equal-frequency binning

PSAV state	Interval	
	Frequency [%]	Value [ng/(ml ·mo)]
*L*_*bq*_	(0, 20]	(, −5.567]
*M* *L*_*bq*_	(20, 40]	(−5.567, −0.611]
*M*_*bq*_	(40, 60]	(−0.611, −0.026]
*M* *H*_*bq*_	(60, 80]	(−0.026, 0.005]
*H*_*bq*_	(80, 100]	(0.005,)

*L*_*bq*_= Low, *M* *L*_*bq*_= Medium low, *M*_*bq*_= Medium, *M* *H*_*bq*_= Medium high, *H*_*bq*_= High

**Table 9:** Before-nadir candidate patterns by equal-frequency binning

Pattern (support)			
HSPC		CRPC	
*L*_*bq*_	(0.78)	*L*_*bq*_	(0.64)
*M* *L*_*bq*_	(0.52)	*M* *L*_*bq*_	(0.61)
*M*_*bq*_	(0.41)	*M*_*bq*_	(0.52)
*M* *H*_*bq*_	(0.36)	*L*_*bq*_→*M*_*bq*_	(0.31)
*H*_*bq*_	(0.37)	*M* *L*_*bq*_→*M*_*bq*_	(0.33)
*L*_*bq*_→*M* *L*_*bq*_	(0.33)		

*L*_*bq*_,*M* *L*_*bq*_,*M*_*bq*_,*M* *H*_*bq*_,*H*_*bq*_= PSAV state (Table 8)
